# Combined Effect of Subchondral Drilling and Hyaluronic Acid with/without Diacerein in Full-Thickness Articular Cartilage Lesion in Rabbits 

**DOI:** 10.1100/2012/310745

**Published:** 2012-04-30

**Authors:** Wanwisa Suwannaloet, Wiroon Laupattarakasem, Peerapol Sukon, Siriwan Ong-Chai, Pisamai Laupattarakasem

**Affiliations:** ^1^Department of Pharmacology, Faculty of Medicine, Khon Kaen University, Khon Kaen 40002, Thailand; ^2^Department of Orthopaedics, Faculty of Medicine, Khon Kaen University, Khon Kaen 40002, Thailand; ^3^Department of Anatomy, Faculty of Veterinary Medicine, Khon Kaen University, Khon Kaen 40002, Thailand; ^4^Department of Biochemistry, Faculty of Medicine, Chiang Mai University, Chiang Mai 50200, Thailand

## Abstract

The osteochondral healing potential of hyaluronic acid (HA) plus diacerein was evaluated in subchondral-drilling- (SCD-) induced fibrocartilage generation in rabbits. A full-thickness chondral defect was created along the patellar groove of both knees and then SCD was subsequently performed only in the left knee. A week later, the rabbits were allocated into 3 groups to receive weekly intra-articular (IA) injection for 5 weeks with normal saline solution (NSS) (group 1) or with HA (group 2 and group 3). Starting at the first IA injection, rabbits were also gavaged daily for 9 weeks with NSS (group 1 and group 2) or with diacerein (group 3). The animals were then sacrificed for evaluation. The newly formed tissue in SCD lesions showed significantly better histological grading scale and had higher content of type II collagen in HA-treated group compared to NSS control. In addition, adding oral diacerein to HA injection enhanced healing potential of HA.

## 1. Introduction

Osteoarthritis (OA) is a degenerative disease that affects synovial joints. Initial lesion can be focal and local, but extensive articular cartilage breakdown can result, which may even produce profound alterations of the subchondral bone. The disease is slowly progressive and related with ageing and weight bearing of the joint. Common locations of OA are the knee and the hip, and pain is the most common presentation aggravated by mechanical loading. In early stage of knee OA, symptoms can be alleviated with analgesics, nonsteroidal anti-inflammatory drugs (NSAIDs), and/or intra-articular (IA) injection of hyaluronic acid (HA) [1, 2]. However, these medications are less satisfactory in late-stage OA, for which the treatment of choice is usually a replacement arthroplasty [2]. However, for a smaller focal articular defect, different approaches are proposed to enhance cartilaginous healing potential. Subchondral drilling (SCD) is one of the techniques frequently used in this condition [3]. By drilling or prickling with an awl, the subchondral bone plate within the chondral defect is penetrated. The defect leads to bleeding and subsequent fibrin clot formation filling the defect and covering the exposed bony surface. Bone-marrow-derived stem cells then migrate into the clot and stimulate fibrocartilaginous repaired tissue. Compared to native hyaline cartilage, the regenerated fibrocartilage is thinner and consists mainly of type I collagen, which is less durable to loading than normal hyaline cartilage, which contains mainly type II collagen [4]. This is a significant drawback of this procedure besides its constraint regarding the limited size of the suitable defect [3].

The inferior biochemical and biomechanical properties of the fibrocartilage in comparison to the normal articular cartilage predispose to the development of degenerative osteoarthritis [3]. Research is on going in an attempt to use adjuvant treatments to improve the quality of the repaired tissue after the surgical procedure, with the goal of producing a more hyaline-like repair capable of stable and long-term function. In this experiment, we aim to study the efficacy of IA injection of HA after SCD comparable with the microfracture technique for the treatment of articular cartilage lesions. In addition, one of the symptomatic slow-acting drugs in osteoarthritis (SYSADOA), diacerein, is added to HA therapy to investigate its additive effect compared to administration of HA alone.

HA, a nonsulfated glycosaminoglycan, is a major component found in the extracellular cartilaginous matrix and in the synovial fluid. This compound is generally produced by chondrocytes, synoviocytes, and fibroblasts. Aggregates formed by HA and aggrecan absorb water molecules into the articular cartilage rendering elasticity of this structure and contribute to shock absorption property of the joint. It not only works as a lubricant and a mechanical barrier but also has analgesic, anti-inflammatory, and chondroprotective effects [5, 6]. An anticatabolic effect of HA has been demonstrated in both in vitro and in vivo models [7, 8]. Intra-articular injection of HA was shown to reduce articular lesions of articular cartilage by inhibiting production of interleukin-1*β* (IL-1*β*) [9], nitric oxide (NO) [10], and metalloproteinases (MMPs) [9] leading to further decrease of cartilaginous matrix degradation. HA also enhances chondrocyte proliferation [11, 12] as well as increases synthesis of the components of cartilage, such as sulfated glycosaminoglycans, chondroitin-6-sulphate, proteoglycan, and type II collagen [13], which subsequently increase hyaline-like cartilage.

Diacerein (9,10-dihydro-4,5-bis(acetyloxy)9,10-dioxo-2-anthracene carboxylic acid) is one of SYSADOA for the treatment of OA [16]. After oral administration, it is rapidly broken down and deacetylated into its active metabolite, rhein [17]. The potential disease-modifying properties of diacerein and its metabolite have been shown in vitro and in vivo models to be primarily due to potent inhibition of the production and activity of inflammatory cytokines and other catabolic cytokines expressed in OA, which are involved in cartilage catabolism and also may induce the apoptosis of chondrocytes [18, 19]. In addition to anticatabolic effect, their anabolic properties are also demonstrated [20, 21]. Both diacerein and rhein stimulate production of cartilage growth factors such as transforming-growth-factor-beta (TGF-beta) [20, 21]. This factor stimulates chondrocyte proliferation and consequently increases synthesis of the components of cartilage, such as collagen and proteoglycan. Several randomized controlled trials have demonstrated beneficial role of diacerein in knee and hip OA [22], with pain-relieving action starting around 4 weeks of therapy that still persists for 1-2 months after discontinuation, indicating possible “carry-over” effect [23]. Recent meta-analyses have found diacerein to be significantly superior to placebo and comparable to NSAIDs but with better tolerability profile [24].

## 2. Materials and Methods

### 2.1. Animal

Three-month-old, male New Zealand White rabbits (2.5–3.0 kg, National Laboratory Animal Center, Thailand) were used in this study. All animals were housed individually and allowed unlimited activity and free access to water and food. This experiment has been permitted by the Ethics Committee for Experimental Animals of Khon Kaen University, Thailand (AE 006/53).

### 2.2. Surgical Technique

Each rabbit was pretreated with intramuscular oxytetracycline (General Drug House Co. Ltd., 20 mg/kg), and the rabbit was fasted for 12–18 hours prior to the operation. The animals were anesthetized with intramuscular injection of ketamine (Calypsol, 15 mg/kg), xylazine (L.B.S Laboratory Ltd. Part., 5 mg/kg), and acepromazine (Phoenix Pharmaceutical, 1 mg/kg). Both knee joints were approached through an anterior medial parapatellar longitudinal incision under sterile conditions. Full-thickness articular defects (FTDs) were created as described by Messner [25] with some modifications. In brief, the approximately rectangular (5 mm × 10 mm) defect was performed along the patellar groove of the femoral condyle with a curette without violating the subchondral bone. Only in the left knee, subchondral drillings were introduced to the defect by using a small drill bit (Chondropick). Four drill holes were made in a row inside the bare subchondral area. The operative field was irrigated clean, and the wound was then closed in layers. Bleeding was not active while making sutures and therefore neither drainage system nor external compression was required. Oxytetracycline (20 mg/kg) was given intramuscularly for 6 days, and the stitches were removed on day 7.

### 2.3. Experimental Procedures

A week after the operation, the rabbits were randomly allocated to three groups, 10 of each, receiving: (1) IA injection of normal saline solution (NSS) + daily oral NSS; (2) IA injection of HA + daily oral NSS; (3) IA injection of HA + daily oral diacerein. The NSS used in group (1) was expected to have only placebo effect and was considered a control, whereas in group (2) and (3), each animal received IA injection of 0.3 mL (10 mg/mL) of HA (Ostenil, Chemedica, Germany, MW of 2 × 10^6^ Da) once a week for 5 consecutive weeks into both knees. For group (3), daily oral administration of diacerein (3.5 mg/Kg BW/day) was added to a course of five IA injections of HA and continued until the end of the experiment.

### 2.4. Tissue Processing and Histological Evaluation

10 weeks after operation, the animals were sacrificed and the operated knees were harvested. Repaired tissue was assessed blindly by three investigators both macroscopically and microscopically. After gross assessment, the specimens were prepared and fixed in 10% (v/v) buffered formalin, decalcified with 10% EDTA/TRIS-HCl (pH 7.4), dehydrated with a series of graded alcohol, and embedded in paraffin. Tissue sections (6 *μ*m thick) were stained with Haematoxylin-Eosin (H&E) for morphologic analysis. The type and healing potential of the tissue were assessed by Dorotka's criteria (Table 1) [14] and International Cartilage Repair Society (ICRS) score (Table 2) [15], respectively. Cell proliferation was accounted by measuring proliferating cell nuclear antigen (PCNA, Dako, USA), and collagen type (Collagen I Ab (ab6308), Abcam, USA; Collagen II Ab-2 (Clone 2B1.5), Neo-markers, USA) was determined using immunohistochemical staining. In brief, immunohistochemistry was performed on rehydrated 6 *μ*m thick sections using antibodies to PCNA (dilution 1 : 400), type I collagen (dilution 1 : 200), and type II collagen (dilution 1 : 100). The antigen retrieval was digested with pepsin (Dako, USA) at 1 mg/mL Tris-HCl, pH 2.0 for 15 min at room temperature in case of collagen I and collagen II. Envision and system-HRP-labeled polymer (Dako, USA) were used to locate immunoreactivity with DAB as chromogen. Contrast staining with hematoxylin was performed followed by dehydration, mounting, and qualitative light microscopic evaluation.

### 2.5. Statistical Analysis

Histological scores were assessed for their normality and equal variance assumptions of parametric statistics. If the data satisfied these assumptions, one-way analysis of variance (ANOVA) was used to compare means of histological scores among treatment groups. If the F-test was significant (*P* < 0.05), then the Scheffe test for multiple comparisons was used to find out which pairs of treatment groups were significantly different. If the data violated the assumptions, they were analyzed by the Kruskal-Willis test and Mann Whitney test. The data were expressed as mean ± standard error of means (SEM). Differences were considered significant if *P* < 0.05. All statistical tests were prepared using SPSS (version 17, SPSS, Inc, Chicago).

## 3. Results

None of the animals showed any evidence of infections or allergic reactions from IA injection of HA.

### 3.1. Morphological and Histological Characteristics of FTD and SCD in Rabbit Knees

**Figure 1:**
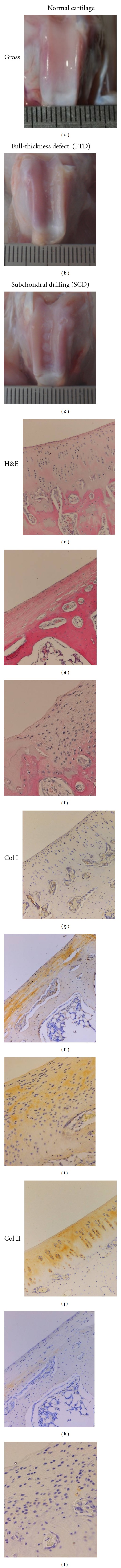
Gross morphology, H&E-stained, and immunohistochemical stained sections of normal (left panel), FTD (middle panel), and FTD plus SCD (right panel) 10 weeks after operation (Col I: collagen I; Col II: collagen II; NSS: normal saline solution; HA: hyaluronic acid; DC: diacerein).

Gross, H&E stained, and collagen type histology from the regenerated cartilaginous tissue after FTD and SCD creation were evaluated (Figure 1).

#### 3.1.1. Gross Morphology

Normal articular cartilage from the rabbit knee aged 3-4 months appeared white, clear, and glistening (Figure 1(a)). After 10 weeks of surgical operation, gross evaluation of the repaired tissue demonstrated that the FTD site appeared dull and pale-yellowish (Figure 1(b)), while the drilling holes within the FTD lesion contained new cartilaginous tissue filling up the lesions (Figure 1(c)).

#### 3.1.2. Histological Characteristics

**H&E Staining**

The histology of normal articular cartilage shows the horizontal lining of superficial chondrocytes along with the articular surface and aligned as vertical columns in deeper zone (Figure 1(d)). According to Dorotka's criteria (Table 1) [14], the repaired tissue showed fibrosis in the FTD lesion (Figure 1(e)), while the SCD induced fibrocartilage formation (Figure 1(f)).

**Collagen Type**

Immunohistochemistry revealed more intense type I collagen in FTD area (Figure 1(h)) compared to SCD (Figure 1(i)) and normal cartilage (Figure 1(g)). In contrast, intensity of type II collagen was found mostly in normal cartilage (Figure 1(j)), which was more than that of FTD (Figure 1(k)) and SCD areas (Figure 1(l)). These findings confirm the fibrosis and fibrocartilage formation in FTD and SCD lesions.

### 3.2. Result of Intraarticular Injection of HA with or without Oral Administration of Diacerein 10 Weeks Following Operation

#### 3.2.1. Histological Analysis

10 weeks after the operation, histological analyses indicated that the quality of the regenerated tissues differed in each group.

**H&E Staining**

**Figure 2:**
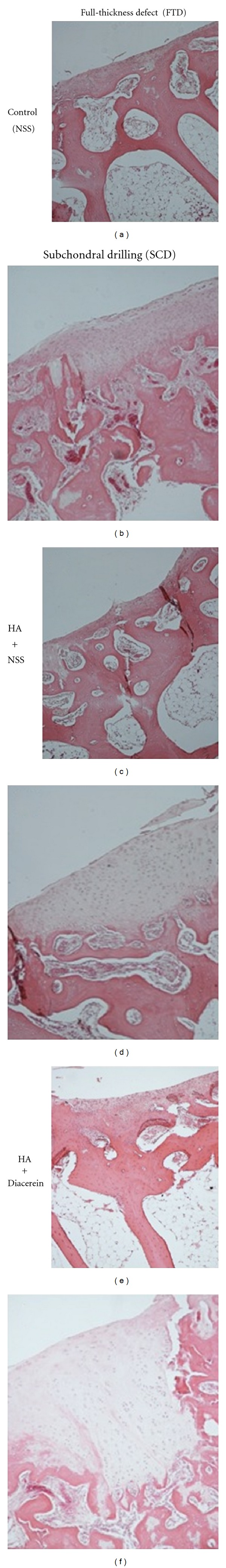
The histological sections of femoral condyle stained with H&E 10 weeks after operation. The sections showed the repaired tissue treated with HA + NSS (c, d) and HA + diacerein (e, f) compared with the control (a, b) (NSS: normal saline solution; HA: hyaluronic acid; DC: diacerein).

H&E staining showed the cartilaginous tissue regenerated markedly in knees with SCD (Figures 2(b), 2(d), and 2(f)) as compared with FTD (Figures 2(a), 2(c), and 2(e)). Furthermore, the repaired tissue in SCD of the animals treated with HA + NSS (Figure 2(d)) or HA + diacerein (Figure 2(f)) was thicker than the control (Figure 2(b)).

**Figure 3:**
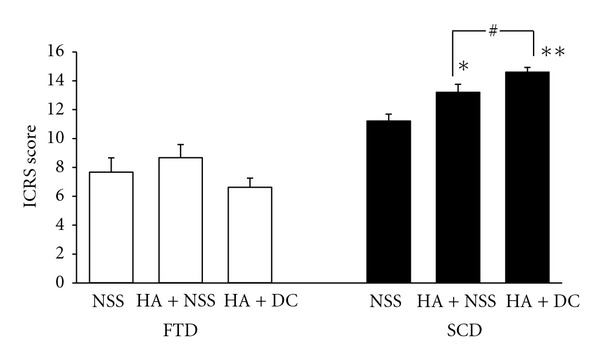
Effect of HA + NSS and HA + DC on ICRS scores for the cartilaginous tissue regeneration of FTD (white bar) and SCD (black bar) 10 weeks after operation. Each value represents the mean ± SEM, *n* = 10. *P < 0.05, **P < 0.001 significantly different from control; ^#^ *P* < 0.05 significant difference between two groups. (NSS: normal saline solution; HA: hyaluronic acid; DC: diacerein; ICRS: International Cartilage Repair Society; SCD: subchondral drilling; FTD: full-thickness articular defects).

Using a modified component of the International Cartilage Repair Society (ICRS) visual histological assessment scale (Table 2) [15], the mean ICRS score for repaired tissue grading of the FTD treated with HA + NSS or HA + diacerein (8.67 ± 0.91 and 6.62 ± 0.64, resp.) was not significantly different from the NSS group (7.67 ± 0.99), while statistical difference was seen in the knees with SCD treated with HA + NSS or HA + diacerein (13.21 ± 0.55, *P* = 0.012 and 14.6 ± 0.33, *P* < 0.001; resp.). Furthermore, HA + diacerein treatment resulted in higher ICRS score compared to HA + NSS treatment (*P* = 0.028) (Figure 3).

**Figure 4:**
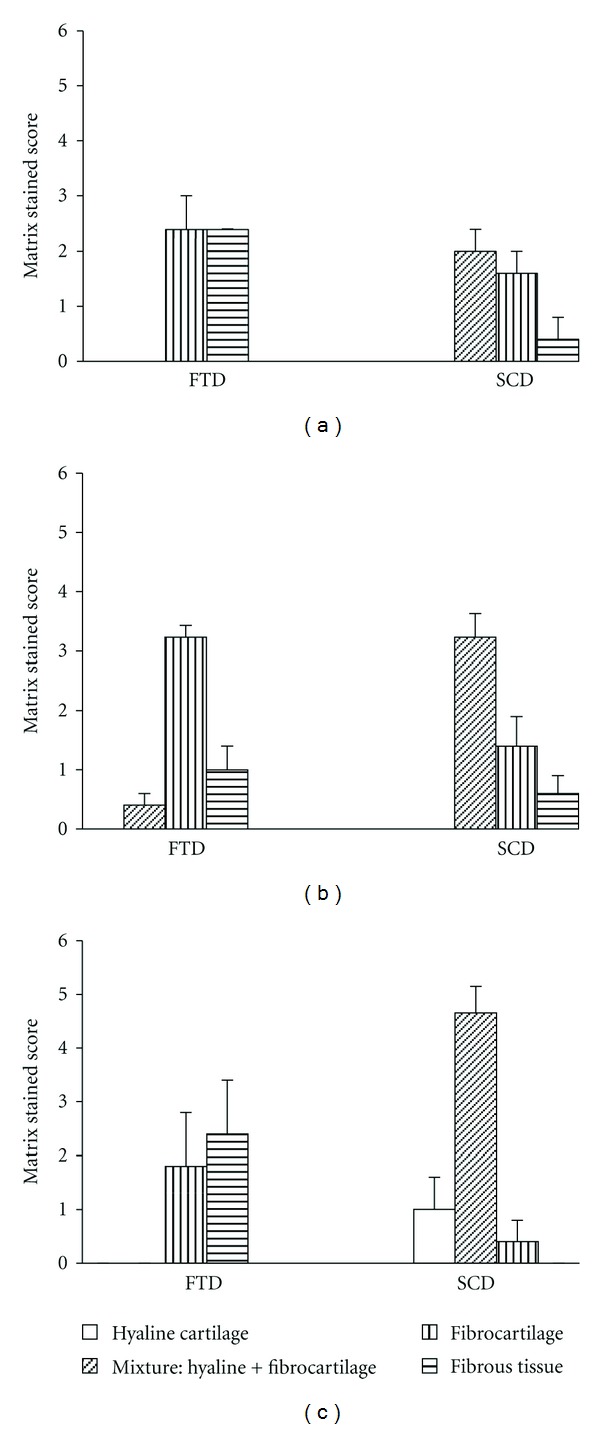
The mean score of the sections of repair cartilage varied according to morphology. Bars represent standard error of the mean (SEM) (NSS: normal saline solution; HA: hyaluronic acid; DC: diacerein; SCD: subchondral drilling; FTD: full-thickness articular defects).

**Figure 5:**
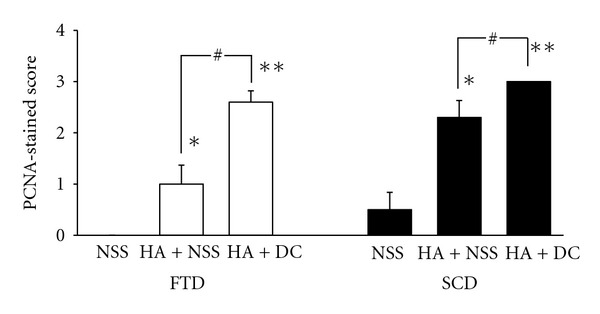
Effect of HA + NSS and HA + DC on cell proliferation, using PCNA staining, 10 weeks after operation. Each value represents the mean ± SEM, *n* = 10. *P < 0.05, **P < 0.001 significantly different from control; ^#^ *P* < 0.05 significant difference between two groups (NSS: normal saline solution; HA: hyaluronic acid; DC: diacerein; SCD: subchondral drilling; FTD: full-thickness articular defects).

Analysis using Dorotka's criteria (Table 1) showed that HA + NSS and HA + diacerein treatments also affected tissue type of the regenerated cartilage. The animal knees with FTD treated with HA + NSS and HA + diacerein showed decrease of fibrous tissue and fibrocartilage compared with the control group, while increase of hyaline type was seen in knees with SCD in HA + NSS and HA + diacerein groups (Figure 4). It is notable that hyaline cartilage formation was observed only in HA + diacerein group. Furthermore, HA + NSS and HA + diacerein treatments also affected cell proliferation (Figure 5).

**PCNA Staining**

Using PCNA staining (Figure 5), cell proliferation was induced in SCD and significantly increased in both HA + NSS- and HA + diacerein-treated animals. Cell proliferation in FTD group was also induced with both treatments. It is noticeable that cell proliferation in both FTD and SCD groups treated with HA + diacerein was significantly higher than that in the group with HA + NSS treatment.

**Collagen Type**

**Figure 6:**
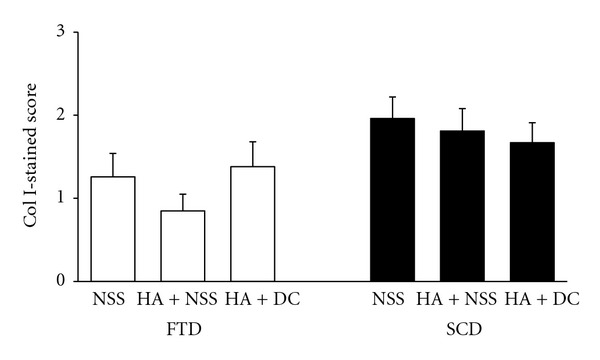
Effect of HA + NSS and HA + DC on type I collagen 10 weeks after operation. Each value represents the mean ± SEM, *n* = 10 (NSS: normal saline solution; HA: hyaluronic acid; DC: diacerein; SCD: subchondral drilling; FTD: full-thickness articular defects).

**Figure 7:**
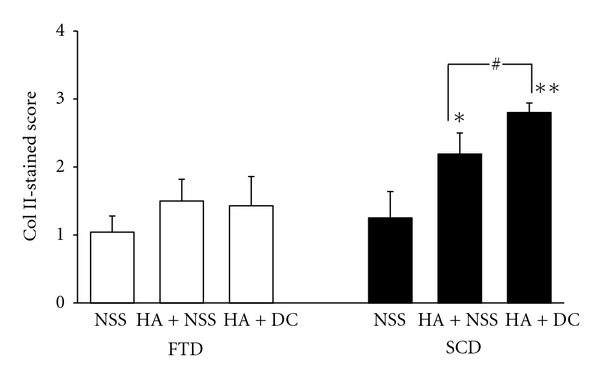
Effect of HA + NSS and HA + DC on type II collagen 10 weeks after operation. Each value represents the mean ± SEM, *n* = 10. *P < 0.05, **P < 0.001 significantly different from control; ^#^ *P* < 0.05 significant difference between two groups (NSS: normal saline solution; HA: hyaluronic acid; DC: diacerein; SCD: subchondral drilling; FTD: full-thickness articular defects).

Using immunohistochemical staining, there was no difference for type I collagen content in animals treated with HA + NSS or HA + diacerein as compared with the controls in knees both with FTD and SCD (Figure 6). The magnitude of type II collagen content for both treatment groups was also not different in FTD, while after SCD, the content was significantly increased in HA + NSS and HA + diacerein treatment groups (*P* = 0.038 and *P* < 0.001, resp.) (Figure 7). It was noted that HA + diacerein-treated group contained significantly higher amount of type II collagen than that treated with HA + NSS (*P* = 0.039).

## 4. Discussion

Friability and mechanical weakness of the fibrocartilage challenge the search of more appropriate substance to modify the quality of the cartilaginous healing. In this experiment, HA with or without diacerein was chosen. Using a modified component of the ICRS cartilage repair assessment scoring scale (Table 2), both HA + NSS and HA + diacerein did not stimulate chondral regeneration in FTD (Figure 3) as had been reported by Evanich [26]. Immunohistochemical staining also showed no difference in both types of collagen contents from FTD in animals treated with HA + NSS or HA + diacerein (Figures 6 and 7). On the contrary, cell proliferation was significantly enhanced by both HA + NSS and HA + diacerein treatments demonstrated by PCNA staining (Figure 5). In addition, cell proliferation in HA + diacerein-treated group was significantly increased compared with the control (*P* < 0.001) or with HA + NSS treatment only (*P* = 0.025). This result suggests that diacerein could promote cell proliferation more than that resulted from HA + NSS treatment. An anabolic effect of diacerein has been confirmed in previous studies [21].

In SCD group, HA + NSS treatment stimulated larger amount of cartilaginous tissue filling in the holes (Figure 2(d)). The tissue was more hyalinelike and contained higher type II collagen content (Figure 7). These results agree with those of Karna et al. who suggested that HA might facilitate prolidase function in collagen biosynthesis [27]. The potential of HA in the repair of injured articular cartilage may be partly accounted by recruiting chondrogenic cells and promoting migration of chondrocytes in the cartilaginous tissue. This finding was also supported by Chung and colleagues [28] by measuring the early gene expression and production of cartilage-specific matrix proteins. Chondrogenesis has been demonstrated in the culture of mesenchymal stem cells (MSCs) in HA hydrogels [29]. MSCs are multipotent progenitor cells released from bone marrow during the drilling procedure and have the ability to differentiate into several cell types, including chondrocytes. Besides MSCs, microfracture also introduces access of many growth factors, such as transforming-growth-factor-*β*1 (TGF-*β*1) and b-FGF, into the joint [30]. These growth factors would then help transform MSCs into chondrocytes and increase chondrocyte proliferation.

The present study also demonstrates the additive effect of diacerein to HA. Significant tissue proliferation, confirmed by the ICRS score (Figure 3) and PCNA staining (Figure 5), was seen at the drilled holes as compared with NSS and HA treatment alone. The additive effect of diacerein to HA might work through production of cartilage growth factors such as TGF-*β* [20]. This factor stimulates chondrocyte proliferation and consequently increases synthesis of components of the cartilage, such as collagen. In addition, an anticatabolic effect of diacerein has also been reported. Both diacerein and its active metabolite, rhein, are powerful inhibitors of inflammatory cytokines, such as IL-1 [31], nitric oxide [18], and metalloproteases (collagenase and stromelysin) [19, 32] that are involved in cartilage degradation. Furthermore, both diacerein and rhein reduce the sensitivity of chondrocytes to IL-1 by reducing IL-1 receptor levels on the cell surface and indirectly increase production of IL-1ra (the natural inhibitor of IL-1receptor binding) in human cartilage through decreased NO production [18]. By inhibiting the IL-1 converting enzyme (ICE) production, diacerein and rhein reduce the production of active IL-1 [33].

In conclusion, our findings suggest that five successive weekly IA injections of HA a week after SCD stimulates chondrocyte division and increases type II collagen content. Furthermore, adding diacerein to HA can enhance chondroprotective effect of HA. We believe that this procedure is feasible to improve properties of the regenerated fibrocartilage, as a result of ordinary healing process, to a quality closer to that of hyaline cartilage. This finding opens a new prospect of combining HA and diacerein to SCD, or comparably microfracture, as a treatment of FTD in the human joint.

## Figures and Tables

**Table 1:** Histological criteria used to demonstrate tissue types (Dorotka's criteria) [14].

Tissue type	Histologic criteria
Fibrous tissue	(i) Highly orientated type I collagen fibrous structure (ii) Cells with elongated shape, not in lacunae

Hyaline cartilage	(i) Ground glass-like appearance of the matrix (ii) Type II collagen staining (with variable intensity of the latter) (iii) Spherical chondrocytes in mature lacunae

Fibrocartilage	(i) Encompassed a wide range of tissues between fibrous tissue and hyaline cartilage, including fibrocartilage (ii) Oval and spherical cell shapes with and without lacunae (iii) Varying intensity of staining for proteoglycans and type I and II collagen (iv) No hyaline appearance of the matrix

Articular cartilage	(i) Hyaline cartilage with columnar arrangement of the cells (ii) Matrix staining typical of articular cartilage

**Table 2:** ICRS Visual Histological Assessment Scale (modified by Mainil-Varlet, 2003) [15].

Feature	Score
H&E	
(I) Surface	
Smooth/continuous	3
Discontinuities/irregularities	0
(II) Matrix	
Hyaline	3
Mixture: hyaline/fibrocartilage	2
Fibrocartilage	1
Fibrous tissue	0
(III) Cell distribution	
Columnar	3
Mixed/columnar clusters	2
Clusters	1
Individual cells/disorganized	0
(IV) Cell population viability	
Predominantly viable	3
Partially viable	1
<10% viable	0
(V) Subchondral bone	
Normal	3
Increased remodeling	2
Bone necrosis/granulation tissue	1
Detached/fracture/callus at base	0
(VI) Cartilage mineralization (calcified cartilage)	
Normal	3
Abnormal/inappropriate location	0
PCNA	
Positive	3
Negative	0
Collagen type I	
Abundant	3
Little	1
No	0
Collagen type II	
Abundant	3
Little	1
No	0
